# THE INTERGENERATIONAL EFFECTS OF THE NEW DEAL WORK RELIEF PROGRAMS ON LATE-LIFE OUTCOMES: AN 80-PLUS-YEAR FOLLOW-UP STUDY

**DOI:** 10.1093/geroni/igz038.2964

**Published:** 2019-11-08

**Authors:** Sepideh Modrek

**Affiliations:** San Francisco State University, San Francisco, California, United States

## Abstract

Evidence from multiple disciplines suggests that early‐life conditions and environments affect outcomes across the life course. However, less is known about the effects of policy interventions targeted to adults and communities that may have intergenerational consequences on exposed children. In this study, we undertake novel data linkages to examine the effects of the New Deal work–relief programs on long-term health, disability and mortality outcomes of children born between 1920-1940. We first link the American Manufacturing Cohort (AMC) workforce backward to their childhood census records to capture parental and community exposure to New Deal work-relief programs. We then test the hypothesis that employment in New Deal work-relief programs is associated with lower levels of chronic disease, lower rates of disability and delayed mortality for both the children in benefitting households and children in non-benefit households living in areas that received greater amounts of New Deal funding.

